# Prevalence of, and barriers to the disclosure of HIV status to infected children and adolescents in a district of Ghana

**DOI:** 10.1186/s12914-017-0114-6

**Published:** 2017-04-08

**Authors:** Eric Gyamfi, Paul Okyere, Acheampong Enoch, Emmanuel Appiah-Brempong

**Affiliations:** 1 Margaret Marquart Catholic Hospital, Kpando, Ghana; 2 Department of Health Promotion and Education, School of Public Health, Kwame Nkrumah University of Science and Technology, Kumasi, Ghana; 3 Department of Community Health, Centre for Disability and Rehabilitation Studies, Kwame Nkrumah University of Science and Technology, Kumasi, Ghana

**Keywords:** Prevalence, Barriers, HIV disclosure, Children, Adolescents

## Abstract

**Background:**

Globally there are about 3.3million children under the age of 15 years living with HIV. Of this number, 88% live in sub-Saharan Africa. In Ghana, an estimated 33,000 children were said to be living with the HIV infection in 2012. Lack of disclosure adversely affects the well-being of the child, including access to paediatric HIV treatment and care and adherence to treatment. However, the greatest psychosocial challenges that parents and caregivers of HIV-infected children face is disclosure of HIV status to their infected children. This study sought to determine the prevalence of and the barriers to the disclosure of HIV status to infected children and adolescents in Lower Manya-Krobo District in Ghana.

**Methods:**

A cross sectional study with a sample of 118 caregivers of HIV infected children and adolescents aged 4–19 years attending three HIV clinics in the Lower Manya Krobo District, and 10 key informants comprising of healthcare workers and HIV volunteer workers involved in the provision of care to infected children and their families.

**Results:**

The prevalence of disclosure was higher. Main barriers to disclosure identified in this study included age of child, perceived cause of HIV, stigma attached to HIV, child’s inability to keep diagnosis to self and fear of psychological harm to child.

**Conclusion:**

There is the need for the Ghana Health Service in conjunction with the Ghana Aids Commission and the National Aids Control Programme to develop comprehensive context-based disclosure guidelines.

## Background

Globally there are about 3.3million children under the age of 15 years living with HIV. Of this number, 88% live in sub-Saharan Africa [1]. In 2011 alone, a total of 330,000 children under the age of 15 years were newly infected, 90% of who live in sub-Saharan Africa [2]. In Ghana, an estimated 33,000 children were said to be living with the HIV infection in 2012 (UNAIDS, 2012).

One of the greatest psychosocial challenges that parents and caregivers of HIV-infected children face is disclosure of HIV status to their infected children, even though there are a myriad of benefits associated with disclosure [3].

The World Health Organisation (WHO) reports however that, disclosure of HIV status to infected children, the gateway to treatment has failed to keep pace with the increasing access to HAART. The WHO also recognizes that the lack of disclosure ultimately affects the well-being of the child, including access to paediatric HIV treatment and care and adherence to treatment. In view of this, the WHO recommends that as part of their long term management, children of school age should be told their HIV status and further recommends that younger children should be told their status incrementally to accommodate their cognitive skills and emotional maturity, in preparation for full disclosure [4]. Similarly, the American Academy of Paediatrics recommends that children of school age and all adolescents should be told their diagnosis. The Academy further stresses on the importance of adopting an individualized approach in the disclosure process. It also suggests that younger children need not know their diagnosis but should have their illness discussed with them [5].

There are differences in the HIV disclosure prevalence rates among countries which have been reported by several researchers. For example, it is reported that in India, the disclosure rate is 14% [6] and 13% in Nigeria [7]. In the study by Feinstein et al. [8], 77% of adolescents older than 11 years had been told their HIV diagnosis. Similarly, in a prospective cohort study, 57% of 10–16 year olds knew that they were infected [9]. The higher disclosure rate observed in the resource rich countries might reflect a better education of the caregivers and understanding of the importance of disclosure.

Disclosure of health status to individuals, especially children is a fundamental human right issue that cannot be ignored. For example, in article 24 of the United Nations Convention on the Rights of the Child, children have the right to good quality health care – the best health care possible – to safe drinking water, nutritious food, a clean and safe environment, and information to help them stay healthy. It is therefore important for parents and caregivers to provide adequate information for children who are HIV positive. Provision of such information would help protect the health rights of these children.

Available data from two independent studies conducted in Ghana and South Africa suggest that very few children living with HIV are actually aware of their diagnosis [10]. These studies reported a prevalence of 9% [11] and 21% [12] respectively. It must however be mentioned that these studies did not explore the barriers to and benefits of HIV status disclosure.

The traditional and cultural orientation of Ghanaians puts individuals, their behaviour and their general health status under the scrutiny of the larger family members. The Ghanaian society is highly traditional and has some level of relatively high communal spirit. Family members do not want others to taint the image of the larger family and would try as much as possible to protect the long standing image within the society. The orientation of Ghanaian communities means that individual behaviour casts a reflection on the larger family and/or community group. In most Ghanaian communities, communal spirit overrides individual aspirations, people are therefore afraid to know their HIV status due to the possible spontaneous negative reaction from members of the society. The reaction may include stigma, labelling and social distance or withdrawal. Ghanaian society has a way of categorising and assigning expectations to its members. When people perceive individuals as possessing socially unacceptable attributes, in this case HIV, they assign negative qualities to the person and this results in intense devaluation of the individual. This traditional approach infringes on the fundamental rights of children and family members in the area of right to dignity. This results in the marginalisation of people living with HIV.

In Ghana, one of the main factors influencing the decision to disclose status of certain sicknesses such as HIV is a desire to protect the family from shame. This is because, in some traditional communities, certain sicknesses are seen as very shameful and are caused by the supernatural when one offends the ‘gods’.

This study therefore aimed to determine the prevalence of disclosure of HIV status to infected children and adolescents and to examine the barriers to the disclosure process.

## Methods

The study was carried out in three hospitals in the Lower Manya-Krobo District of the Eastern Region of Ghana that offer paediatric HIV care. These hospitals were the Atua Government Hospital, the St. Martins de Porres Hospital and the Akuse Government Hospital. A cross sectional design employing a mixture of quantitative and qualitative research methods was used. The advantage of using a mixed method approach was to gain a better and thorough understanding of the subject.

The quantitative aspect of the study involved the administration of structured questionnaires to respondents comprising caregivers of HIV infected children and adolescents aged 4–19 years attending the three hospitals. One hundred and eighteen(118) caregivers were conveniently sampled within a period of 3 months. Caregivers who had come to the hospital with their children at the times of our visits were approached and the purpose of the study was explained to them. Those who were readily willing to participate in the study were given the questionnaire to fill. Respondents were asked questions such as ‘what in your view is the main barrier that prevents you as a caregiver from disclosing the HIV diagnosis to your infected children?

In order to allay respondents’ fear of inadvertent disclosure of an infected child’s status, children were not interviewed at all. For this same reason, caregivers were not interviewed. The qualitative aspect of the study occurred within the same period and it included in-depth interviews with ten (10) purposefully selected key informants comprising mainly of healthcare workers and HIV volunteer workers involved in the provision of care to infected children and their families. The interviews were conducted by three research assistants who were recruited and trained by the researchers. The interviews were conducted at the hospital premises for the caregivers and for the healthcare workers, it took place at their offices. The interviews were conducted in Twi language (Local language). The interviews were tape-recorded with audio recorder together with notes that were taken during the interaction period. Transcripts were coded on dominant themes that were developed from the responses which were given by participants, and were analysed in duplicate, with each analyst blind to the summary of the other. Coding was undertaken by a team of 3research assistants under the supervision of the researchers. Members were paired to read a selected number of transcripts, and both members in each pair read all transcripts independently. The entire team then converged to discuss emerging themes and to resolve disputes on coding. One team member read all the transcripts and facilitated the discussion. The division of transcripts into sections helped in the management of the data. The team compared summaries and involved extra readers to resolve any differences. Data-led approach thematic analysis was used to analyse the data after transcribing the interviews verbatim.

### Limitations of study

In this study, disclosure was based on the subjective reporting of the caregivers and consequently might therefore not reflect the child’s perception. Another limitation of the study could be related to the use of convenience sampling technique which is prone to bias with a possibility of the sample not being representative.

## Results

### Quantitative component

#### Socio-demographic characteristic of caregivers and healthcare workers

Table 1 presents the socio-demographic characteristics of caregivers who participated in the study. The mean age of the caregivers was 48 years. Majority (55.9%) of the caregivers were HIV positive. Most of the caregivers (47.5%) were the child’s biological mother and only 3.4% of them had tertiary level education.

**Table 1:** Socio-demographic characteristic of caregivers and Healthcare workers

Variables	Characteristics	Frequency (n = 118)	Percentage (%)
Age of Caregiver	▪ 21–30	7	5.9
	▪ 31–40	33	28.0
	▪ 41–50	34	28.8
	▪ > 50	44	37.3
	*Mean (SD); Min/max*	*48 (14.02); 21/85*	
HIV status of caregiver	▪ Positive	66	55.9
	▪ Negative	40	33.9
	▪ Unknown	12	10.2
Relationship with the child	▪ Mother	56	47.5
	▪ Father	9	7.6
	▪ Grandparent	31	26.3
	▪ Other	22	18.6
Occupation of Caregivers	▪ Unemployed	11	9.4
	▪ Farming	19	16.2
	▪ Trading	487	41.0
	▪ Artisanship	18	15.4
	▪ Public	13	11.1
	▪ Private	8	6.8
Age group of healthcare workers	• 20–29 years	2	20.0
	• 30–39 years	5	50.0
	• 40 years and above	3	30.0
Status within the hospital	• Medical Doctor	4	40.0
	• Nurse	3	30.0
	• Volunteer/Counsellor	3	30.0
Number of years worked in the hospital	• Less than 5 years	2	20.0
	• 5–9 years	6	60.0
	•10 years and above	2	20.0

In terms of healthcare workers, 50% were between the ages of 30–39years, 30% were either exactly 40 years or more while 20% were below 30 years. Regards to their status within the hospitals, 40% were medical doctors, 30% were nurses while another 30% were volunteers or counsellors who were working with HIV patients. About 60% of the healthcare workers had worked with the HIV unit between 5 and 9 years, 20% had worked for 10 years or more and 20% had worked less than 5 years.

The characteristics of the children whose caregivers participated in the study are presented in Table 2. The average age of the children was 11 years. The results revealed that most (36.8%) of the children were first diagnosed with HIV from ages 6 – 10 years. The majority (94.1) of the children were on antiretroviral medication whereas only 5.9% were not on antiretroviral medications and most (94.9%) of the children were in school.

**Table 2:** Characteristics of Children whose caregivers took part in the study

Variables	Frequency	Percentage (%) (*n* = 118)
Age of Child		
▪ 4–6	24	20.3
▪ 7–11	33	28
▪ 12–16	45	38.1
▪ 17–19	16	13.6
*Mean (SD); Min/max*	*11 (4.31); 4/19*	
Age of child at first diagnosis with HIV		
▪ Less than a year	6	5.3
▪ 1–5 years	12	10.5
▪ 6– 10 years	42	36.8
▪ 11–15 years	33	28.9
▪ 16–18 years	21	18.4
Child on Antiretroviral medication		
▪ Yes	111	94.1
▪ No	7	5.9
Child Currently in School		
▪ Yes	112	94.9
▪ No	6	5.1
Level of Education of Child		
▪ Kindergarten	10	9.2
▪ Nursery	7	6.4
▪ Primary	63	57.8
▪ JHS	29	24.8
▪ SHS	2	1.8
Age at disclosure for children who have been told (*n* = 39)		
▪ 7–11	3	7.7
▪ 12–16	24	61.5
▪ 17–19	12	30.8

#### Prevalence of disclosure of HIV status to infected children

The prevalence of disclosure of HIV status to infected children is presented in Table 2 the majority (66.7%) of the children had not been told their HIV positive status. The results also showed that 89.7% of those who had not disclosed indicated they had intentions of telling their children their HIV positive diagnoses in the near future.

#### The barriers to disclosure of HIV status to infected children

The barriers to disclosure of HIV status to infected children are presented in Table 3. The study elicited views of caregivers about telling the child his/her HIV status. Most respondents (77.7%) had positive views about disclosing the HIV status of an infected child to him and the majority (43.2%) of the respondents reported the preferred age of disclosure of HIV status to infected children to be between 14 and 17 years. With regard to the barriers to disclosure, majority (67.7%) reported that the child might be too young to understand the information. Caregivers however did not provide reasons to why they thought their children might be too young to understand the information.

**Table 3:** The barriers to disclosure of HIV status to infected children

Variables	Frequency	Percentage (%)
Opinion about telling a child his/her HIV status	(*n* = 118)	
− Positive	87	77.7
− Not sure	3	2.5
− Negative	27	22.9
Preferred age of disclosure		
− 6–9	5	4.2
–10–13	33	28.0
− 14–17	51	43.2
− 18–19	29	24.6
*Mean (SD); min/max*	*14.9 (3.01) 6/19*	
Face barrier in telling the child his/her HIV status		
− Yes	93	79.1
− No	25	20.9
Barriers to the disclosure of status to child (*n* = 105)	6	9.2
− Fear of psychologically harm to child	44	67.7
− Child may be too young to understand	12	18.5
− Child will not be able to keep the diagnosis	4	4.6
− Other		

#### Qualitative components

The qualitative components of the results of the study have been presented under the sub-themes which emerged from the analysis of the data. The qualitative component of the study explored the barriers to disclosure process from the perspective of those who provide paediatric care to the caregivers and their children. Four main themes emerged from the data concerning the barriers that prevent caregivers from disclosing the status of their infected children/adolescents to them: age of child; fear of the aftermath of disclosure; need for disclosure; and inability to get child to keep his/her diagnosis to him/herself.

#### Age of Child

Age of the child came up as a very important barrier to disclosure. Most caregivers considered children younger than 13 years as immature and incapable of understanding their illness. According to a trained counsellor and a health care provider at one of the hospital’s HIV Testing and Counselling unit “Some of the children are under aged especially those under 13 years”. Similarly, another trained counselor and a health care provider in another HIV Counseling and Testing unit of one of the hospitals indicated that most of the children are considered too young to be able to understand their illness”

*“Some of the caregivers may find the children too young to understand. Also most of the children have lost one or both of their parents so by disclosing you may end up also telling the children about some other things which they don’t want them to know—such as the death of their parents. They therefore want to wait till the child is old enough to understand and take in all of these other issues before they tell them”* (male caregiver individual interview).

#### Fear of aftermath of disclosure

Generally, the possibility of some undesired consequences arising out of disclosure served as an important barrier to disclosure. Some of these possible undesirable consequences include the effect of disclosure on mother-child relationship, child’s emotional and psychological wellbeing and the perception of mothers that their children may see them as having led irresponsible lives to have contracted the infection

*“…Caregivers are afraid that after disclosure, something bad may happen to them or to the children. For instance the child may refuse his/her medications…if he or she has entered his/her teenage years may blame their caregiver (mother) for infecting him/her with the disease. The child may even get angry and leave home” (*HIV Counsellor, individual interview).

*“Others are also scared of aftermath of the disclosure. They fear something bad might come out of the disclosure. Telling the child of the condition and explaining to the child how the infection came about they are not sure what will happen…they are scared that the child may distant himself/herself from the parents or may even stop taking their medication. Some may even begin to have suicidal ideations.”* (Physician assistant and a trained HIV counselor, individual interview).

#### Stigmatization of HIV

Another major barrier to disclosure of HIV status was the stigma associated with the illness. They were of the view that in their communities, HIV is seen as sickness caused by the supernatural powers as a result of either punishment or breaking communal taboos. Once people get to know of your HIV status, you are labeled and people will not be willing to interact with you. This will affect people’s participation in community activities. In order to avoid these negative attributions and labeling, caregivers decide not to disclose the status to the children.

*“People still perceive HIV as something caused by the spirits. Because of this, the whole community will withdraw from you if they become aware of your status as positive”* (Health service provider, individual interview).

#### The need for disclosure

For a number of caregivers, there is just no need to disclose to the child his/her HIV positive status. Such caregivers were said to be either ignorant or not motivated to disclose to the child.

*“Some of the caregivers also don’t deem it necessary to disclose to the children who don’t ask questions about reason for medication and who are adherent to their medication regimen”*(Trained counselor and a health service provider, individual interview).

#### Inability to get child to keep his diagnosis to him/herself

The stigma attached to HIV and the attitude of children as ‘free talkers’ makes it difficult to make it known to the children their HIV positive status. Study participants were of the view children are more likely to disclose their status either wittingly or unwittingly to their friends through conversations and were wary of the consequences should this happen.

*“They may also not be able to keep the diagnosis to themselves. Because of the stigma attached to the condition, they want to make sure that the child is old enough to keep the diagnosis in order not to let out family secrets.”*(Volunteer worker at an HIV Testing and Counseling Unit, individual interview).

*“Telling children can be problematic because children talk a lot. They may actually disclose the diagnosis to others. This will reveal the status of the mother and the rest of the family, which can result in stigmatization towards them”* (Trained counselor and health service provider, individual interview).

## Discussion

The study sought to determine the prevalence of disclosure and to examine the barriers that caregivers face during disclosure using a mixed method approach. The prevalence of disclosure to children with HIV in this study was 33.3%. The higher prevalence rate observed in this study might be attributed to the increased number of adolescents (mean age of 11, range 4–19 years) in this study. However even when compared with studies involving adolescents and older children, the prevalence obtained in the current study is still very low. In the study by Feinstein et al. [8], 77% of adolescents older than 11 years had been told their HIV diagnosis.

In this study, even though most caregivers agreed that disclosure was important, they preferred to wait till the children were older before disclosing to them their diagnosis. The preferred age of disclosure ranged from 6 to 19 years with a mean age of 14.9 years.

Consistent with findings of the quantitative component of the study discussed above, the qualitative component revealed a generally low rate of disclosure in the district. Additionally, this component also revealed that not only do caregivers like to wait till early teens before disclosure, but also like to wait till they are either prompted by the health care worker or until the child begins to refuse medications on the grounds that they don’t feel sick and therefore do not see the need to take medications daily. This is similar to findings in a study by Hammami et al. [13] which suggested that sometimes children do not understand why they have to take medications when they don’t feel sick.

Under these circumstances caregivers and health care workers are forced to disclose to older children. For children considered too young, caregivers and health care workers try to provide just enough information often without mentioning the name of the disease to enable such children take their medications. This approach to disclosure often referred to as partial disclosure in which bits of information are given out to children without mention of the name of the disease has been used by some caregivers as reported in a number of studies including those conducted by Brown et al. [7] and Oberdorfer et al. [14].

This study revealed that most caregivers face barriers when it comes to the disclosure of HIV status to their infected children. The most commonly mentioned barrier to disclosure according to caregivers was that the child was too young to understand his/her illness. A qualitative component of this same study further lends support to this finding reporting that most caregivers considered children below the age of 13 years as being incapable of understanding their illness. A perception which is even worse for children below the age of 10 years who according to respondents are not told their diagnosis at all.

The second most commonly cited barrier from this study was the fear that children would not be able to keep their diagnoses to themselves, another finding which is common to both the quantitative and qualitative components of the study. However, the failure of the caregivers to disclose the child’s HIV status to him/her may also stem from their own desire to avoid stigmatisation when it becomes known that they are also HIV-infected. This may be a plausible reason for non-disclosure from the perspective of the caregivers since HIV and AIDs still remains highly stigmatised condition in Ghana.

In most Ghanaian communities, communal spirit overrides individual aspirations, people are therefore afraid to know their HIV status due to the possible spontaneous negative reaction from members of the society. The reaction may include stigma, labelling and social distance or withdrawal. Ghanaian society has a way of categorising and assigning expectations to its members. When people perceive individuals as possessing socially unacceptable attributes in this case HIV, they assign negative qualities to the person and this results in intense devaluation of the individual. In order for children not to be stigmatised and excluded from mainstream society, caregivers resort to concealing the HIV status of children. The strategies adopted by caregivers in this Ghanaian society is consistent with the theory of stigma propounded by Goffman [15] that there is always deliberate concealment of information about the stigmatising attribute when it is not immediately visible or known by others. Under such situations management of information about the stigmatising attribute becomes a central issue. This is because all humans will like others to see and interact with them as normal individuals in society,

Again, the cultural interpretation of diseases such as HIV/AIDS in Ghana might have also influenced the decision of caregivers not to disclose the HIV status of the children. In most traditional communities in Ghana, HIV is still seen as spiritual sickness inflicted by the supernatural as a result of sins committed. Such stigmatising cultural meanings can have serious impact on the illness experience of patients and can worsen the social suffering of the individual even more than the symptoms of the disease As a result people hesitate or choose not to disclose their status to family members, friends or neighbours for fear of being ostracised. The belief about the transmission and curability of HIV/AIDS is a reflection of the way Ghanaian society understands the disease.

In addition, the caregivers indicated that fear of psychological and emotional harm to the child is a barrier to disclosure. This is because, HIV/AIDS is a condition that evokes shame and condemnation across many societies in Ghana and consequently could result in traumatic experience such as depression and harmful behaviour in children and or adolescents when they learn that they have contracted this infection. Perhaps, it is in view of this that caregivers fail to disclose the HIV status to their children in order to avert the psychological, emotional and social consequences that may attend disclosure.

Finally, one of the reasons less commonly referred to for non-disclosure in this study were caregivers’ feeling of shamefulness about their illnesses, fear of rebellion from older children for passing on illness to them and caregivers’ perceived lack of the know-how for disclosure. This finding is consistent with a study by Sirikum et al. [16] which reported that most caregivers simply did not know how to disclose and therefore relied on the health care worker to disclose to their infected children.

Another set of barrier was the discretion of caregivers based on their fears of the possibility of undesirable consequences following the aftermath of disclosure and the fact that they saw no need to. Key among the numerous fears expressed by caregivers was the anticipation that children would ask how parents got infected and that answering such a question would compel biological caregivers to give detailed explanation of their sexual activities in an attempt to explain the nature of the illness to the child.

With respect to need for disclosure, this component of the study revealed that caregivers only saw the need to disclose if children were having troubles adhering to their medications or if they asked probing questions about their illness or medications. For parents who argue along this line, it may seem that the only reason they may have for disclosing to the child is to make him/her adhere to the medications. There is therefore the need for health care workers to educate caregivers about the several other benefits that are associated with disclosure. This will provide adequate reason for caregivers to disclose to their children even if children are neither refusing medications nor asking probing questions about their illness.

## Conclusion

The data obtained from this study suggests a low prevalence of disclosure of HIV status to infected children in the Lower Manya-Krobo district of the Eastern region of Ghana. Among the several barriers identified in the study are the age of the child, the fear that the child might not keep the diagnosis to self and the fear of emotional and psychological harm to the child. Given that all the above-mentioned barriers are child-related, the data appears to suggest that caregivers delay HIV disclosure in an attempt to protect their infected children. However, in reality, HIV disclosure to children is delayed to a large extent to protect the caregivers from their personal fears such as fear of blame from children that could mar their relationship with the child as well as fear of having to answer questions about past sexual behaviour. Caregivers’ perception that disclosure may be needless when children are apparently taking medication and not asking probing questions about their illness should serve as a basis for health care workers to step up the provision of information and education about the possible benefits of disclosure.

### Recommendations

There is the need for the Ghana Health Service in conjunction with the Ghana Aids Commission and the National Aids Control Programme to develop comprehensive context-based disclosure guidelines.

Again, it is recommended that stakeholders should adopt the recently released WHO guidelines to the local context of the caregivers. Such guidelines should be part of a comprehensive care programme that focuses on improving support for caregivers and infected children during disclosure.

## Abbreviations

AIDS: Acquired immunodeficiency syndrome
HAART: Highly active antiretroviral therapy
HIV: Human immunodeficiency virus
WHO: World health organization
